# When and how do non-human great apes communicate to support cooperation?

**DOI:** 10.1098/rstb.2021.0109

**Published:** 2022-09-12

**Authors:** Alicia P. Melis, F. Rossano

**Affiliations:** ^1^ Experimental Psychology, University College London, London WC1H 0AP, UK; ^2^ Department of Cognitive Science, University of California San Diego, San Diego CA 92093, USA

**Keywords:** cooperative problem-solving tasks, communication, coordination, great apes, tolerance

## Abstract

Several scholars have long suggested that human language and remarkable communicative abilities originate from the need and motivation to cooperate and coordinate actions with others. Yet, little work has focused on when and how great apes communicate during joint action tasks, partly because of the widely held assumption that animal communication is mostly manipulative, but also because non-human great apes' default motivation seems to be competitive rather than cooperative. Here, we review experimental cooperative tasks and show how situational challenges and the degree of asymmetry in terms of knowledge relevant for the joint action task affect the likelihood of communication. We highlight how physical proximity and strength of social bond between the participants affect the occurrence and type of communication. Lastly, we highlight how, from a production point of view, communicators appear capable of calibrating their signalling and controlling their delivery, showing clear evidence of first-order intentionality. On the other hand, recipients appear to struggle in terms of making use of referential information received. We discuss different hypotheses accounting for this asymmetry and provide suggestions concerning how future work could help us unveil to what degree the need for cooperation has shaped our closest living relatives' communicative behaviour.

This article is part of the theme issue ‘Revisiting the human ‘interaction engine’: comparative approaches to social action coordination’.

## 1. Introduction

The *interaction engine hypothesis* proposes that humans are endowed with a special predisposition for social interaction that is at the basis of language evolution and constitutes the building blocks of our social life and human social institutions [1]. It suggests that humans differ from other primates in the amount of time and effort spent interacting with others, proposing a fundamental difference between humans and other species in terms of motivation to communicate with conspecifics. This hypothesis argues that three ingredients are critical for human communication: (i) attribution of intention (so that behaviours are mapped onto goals); (ii) mutual salience for the participants (common ground, critical for mental coordination); and (iii) Gricean intentions (the goal of having intentions recognized). Grice [2] famously suggested that meaning in a communicative encounter can ultimately be reduced to intention recognition. It is not sufficient for signaller S to have a goal or desire and produce a signal aimed at inducing a response in the recipient, R. It is critical for S that R recognizes S's intention and acts accordingly.

Several scholars subscribe to the idea that human communication, differently from other non-human primates, has a fundamentally ostensive–inferential structure, which relies on the constant computation of relevance of each behaviour in context and an attempt to attribute and read intentions [3,–5]. Other scholars have countered that non-human primate communication shows clear evidence of intentionality and to some degree the equivalent of Gricean intentions (see e.g. [6–9]). Notably, besides his theory of meaning and intentionality, Grice [10] also put forward the notion of a cooperative principle driving human communication, which suggests that all human signals are usually interpreted with the underlying assumption that they have been produced with a cooperative intention, i.e. not to deceive or mislead. This assumption facilitates the inferential process necessary to interpret what a signaller is trying to communicate and what kind of response would be the most appropriate next.

Yet, most animal communication models (which usually exclude humans) assume that manipulation is the driving motivation to communicate [11] or at the very least that the driving force is an attempt to influence the recipient [12] into producing a specific response for the signaller's benefit, rather than assuming a prosocial motivation from the signaller. Once combined with the evidence that great apes appear to perform significantly better in competitive tasks compared to cooperative ones [13,14] and the detection of major tolerance constraints on their ability to cooperate [15], a bias emerged in the study of primate cooperation and communication. The widespread assumption is that little communication is likely to occur during tasks aimed at eliciting cooperation or coordination and that if observed, it would mostly be manipulative in nature and as such resisted (not responded to in alignment with the intentions/goals of the communicator).

Investigating non-human primates' communication in the service of cooperation can cast light on the flexibility of their communicative abilities, as well as the socio-cognitive skills supporting their cooperative behaviour. Furthermore, it can provide new insights into the phylogenetic roots of human cooperation and communication. Indeed, according to the interdependence hypothesis [16], it was the need to coordinate with others in stag-hunt type contexts (i.e. collaborative foraging that allowed individuals to capture preys otherwise unattainable independently) that created high interdependence between individuals and pushed forward the evolution of humans' unique cooperative and communicative skills. Mutualistic collaboration and coordination were the challenge and communication helped solve that challenge.

Naturalistic observations suggest that some degree of interdependence and the need to coordinate in specific contexts might affect the use of communication in non-human great apes. For example, chimpanzees hunt other mammals, and several types of vocalizations occur during this joint activity. Mitani and Watts [17] report on ‘hunting calls’ at the beginning of a hunt to mobilize other chimps to spring into action. Boesch [18] describes ‘hunting barks’ that achieve the effect of informing about a chimpanzee's location, recruiting collaborators and facilitating further coordination, and a ‘capture call’ that conveys the successful completion of a hunt. While not occurring in all communities nor during each hunt, they suggest that the need to galvanize others into action and recruit them for the joint task could be one key motivation to communicate.

Interestingly, male chimpanzees can also produce ‘rest hoo’—vocalizations at the end of a resting session. These vocalizations seem to extend the resting session further, improving social cohesion between individuals and functioning as an alternative to tactile-based bonding [19]. Moreover, a recent study [20] comparing chimpanzees and bonobos on their likelihood of informing others of danger via vocalizations found that chimpanzees were more likely to call and produced more alarm calls when they had not heard a call, contrary to bonobos. The claim is that these differences in motivation to cooperate were due to a higher degree of interdependence in chimpanzees.

Beyond vocalizations, recent work on how great apes get into and out of joint activities [21–23] has shown that they can flexibly select gestural signals to communicate a desire to start or end a joint activity such as grooming or playing, or to resume an ongoing activity that has been interrupted. The occurrence of communicative signals is inversely correlated with bondedness (less communication to start and end joint activities between friends), at least in bonobos. The use of communication to engage in joint activities suggests that beyond interdependence, another important motivator is social cohesion [19].

In this paper, we investigate the evolutionary origins of how communication can be used to facilitate coordination in cooperative tasks by reviewing the current empirical evidence in great apes. Our main focus is on experimental approaches that have investigated collaborative activities in which individuals work together to achieve a common goal, but we also review helping behaviour, in which one individual performs an action to help another one.^1^ While naturalistic observations are key for our understanding of communication in context, we focus on experimental studies because they present both a challenge and an opportunity. The challenge comes from the fact that despite many studies on non-human apes' collaboration and helping, our understanding of their communicative abilities for the purpose of cooperation is still very limited. The opportunity stems from the fact that, via experimental manipulation, specific factors that affect the emergence of communication such as relationship between individuals, inability to solve the task solo, type of information needed to perform the task, can all be controlled, contrary to more naturalistic settings. This provides us with a less noisy landscape to investigate the current questions about how and when great apes communicate in cooperation tasks.

In reviewing the current evidence, we have paid attention to three specific situational challenges inspired by a recent discussion of coordination tasks in humans [25] and we ask *what* it is that needs to be conveyed to achieve success in different cooperative problems.

In the ‘common information’ scenario, when the information necessary to perform the task is perceptually retrievable and available to all participants, coordination can be achieved without communication. Yet, if an individual delays engagement in the joint task (e.g. because of lack of motivation) communication might occur to mobilize them into action, not to tell them what to do. In a scenario of ‘asymmetric attentional focus' there is asymmetry in terms of awareness of what the task is about or what object needs to be acted upon (e.g. in humans informing another participant that the object that needs to be moved is the couch). Individuals are knowledgeable about the specific action required but partners' attentional foci are not necessarily aligned, as it may be the case in pure coordination dilemmas with several possible solutions. In this case, we expect communication aimed at directing the attention of the recipient towards specific objects.

Finally, the scenario of ‘asymmetric knowledge’ is probably the most challenging one, because there is an asymmetry in terms of the information individuals have for the completion of the task, e.g. one individual has visual access to which box has a reward, while the recipient has no access to this information. In this case, some kind of communication to direct the partner to the right location/solution would be needed.

In reviewing the existing evidence, we also pay attention to factors possibly affecting the emergence and type of communication observed (e.g. proximity and mutual visual access of communicator and recipient during the task, relationship between partners, social bonding, e.g. [23]).

## 2. Communication to support cooperation

Scholars investigating human communication tend to break down the main challenge of communication in terms of two components: (i) getting the attention of the relevant individual (this is usually via what have been called ‘summons’, like calling someone's name [26,27]) and (ii) conveying what you need or want (e.g. that you need assistance for a task via ‘requests’ or ‘recruitments’ [28,29]). In research with non-human primates, the first component has often been referred to as ‘attention-getters’ (e.g. producing noise by banging something on the ground to attract attention [30,31]), while the second one either as ‘intention-movement signals' [30,32] (i.e. a signal that is usually the first in a recognizable sequence of behaviours that conveys the intention/goal of the animal, like stretching an arm towards an object can convey an attempt to reach something) or simply as a ‘request’ for something (e.g. a begging gesture used to request for food or a tool; e.g. [33]).

Concerning attention-getters, some scholars have claimed that they are not as critical for great apes, because there is evidence showing that chimpanzees would rather move in front of a human than use attention-getters to attract their attention (e.g. [34]). On the other hand, we know that great apes do produce attention-getters in interaction with conspecifics [35,36] and that they tend to produce visual, often silent, gestures (e.g. a begging gesture) when the recipient is looking at them, while auditory and tactile gestures are more frequent when the recipient is not attending to the signaller. It is also known that the tactile modality is the main modality infant primates use and that visual signals appear later in development [37,38]. In line with the idea that attention-getters are produced first to obtain a recipient's attention, tactile and auditory gestures are the most frequent first gestures in a sequence of gestures [39].

Intention movements are ‘evidence-rich’ behaviours [40] in that they are usually the first step of a known sequence of actions, making recognizability of their meaning often quite straightforward. Recruitment to join an activity can occur both via vocalization (see the calls during hunting previously described) and gestures (e.g. beckoning gestures in bonobos, [41]). Requesting signals do not need to be iconic or part of an action sequence (see e.g. the spin gesture produced by a baby bonobo towards mother to request to be picked up and carried in Halina *et al*. [42]) but they often are (see e.g. the relationship between begging gestures and receiving/catching food from another individual in [33] or the gestures used by bonobos to invite a partner to engage in sex [41,43].

In what follows, we review when communicative signals occur in different experimental studies aimed at eliciting cooperation between participants (table 1), to better understand which factors are more likely to elicit communication and aid coordination.

**Table 1.** Challenges and types of communication observed in cooperative problem-solving studies with non-human great apes. Although there are more studies examining cooperation and coordination abilities in great apes, our focus here is only in those studies that either report or specifically investigated communication aimed at coordination with the partner.

scenario	communication challenge	communication type	partners’ characteristics	references
common information	partner unmotivated/not interested/ inactive	tactile recruitment between conspecifics (partners in the same space)	juvenile chimpanzees	Crawford [44]
			juvenile chimpanzees	Menzel [45]
			chimpanzees: adult male–female infant	Chalmeau [46]
			mother–offspring orangutans	Völter *et al*. [47]
			chimpanzees: adult male–male juveniles	Schweinfurth *et al*. [48]
		attention-getters between conspecifics (distal set-up)	various pairs of unrelated chimpanzees	Voinov *et al*. [49]
		vocalizations and tactile recruitment of human partner (partners in the same space)	young female chimpanzee–human experimenter	Hirata & Fuwa [50] Hirata, Morimura and Fuwa [51]
		no communication	chimpanzee–human (caregiver)	Warneken *et al*. [52]
			various pairs of unrelated chimpanzees	Povinelli & O'Neil [53]
asymmetrical attention	partner's attention directed somewhere else	attention-getters at the desired location (distal set-up)	various pairs of unrelated chimpanzees	Bullinger *et al.* [54]
				Bullinger *et al*. [55]
				Duguid *et al*. [56]
	partner inattentive or inactive	attention-getters between conspecifics (distal set-up)	various pairs of unrelated chimpanzees	Warneken *et al*. [57]
				Melis *et al*. [58]
		attention-getters and visual gestures (proximal set-up)	mother–offspring and unrelated chimpanzee pairs	Yamamoto *et al*. [59,60]
asymmetric knowledge	communicator needs tool from conspecific partner	communication with lexigram keyboard	two chimpanzees previously trained with humans in their roles	Savage-Rumbaugh *et al*. [61]
	communicator needs the human partner to find the tool	pointing gestures (distal set-up)	orangutans–human bonobos–human	Zimmermann *et al*. [62]
			chimpanzees–human	Bullinger *et al*. [63]
	communicator needs the partner to find the food	pointing gestures (distal set-up)	male orangutan–orangutan females male orangutan–human experimenter	Moore *et al*. [8]
	communicator needs a specific tool to perform her role	visual gestures by stretching hand towards tool (proximal set-up)	various pairs of unrelated chimpanzees	Melis & Tomasello [64]
	communicator needs the partner to find the tool	attention-getters at the tool location (distal set-up)	various pairs of unrelated chimpanzees	Bullinger *et al*. [55]
	communicator needs the partner to find the tools and give her one	approach towards and sitting next to the tools' location while offering the key needed to extract the tools. (proximal set-up)	various pairs of unrelated chimpanzees	Melis & Tomasello [65]

### (a) Common information

The majority of the studies on cooperation in great apes have used joint action^2^ tasks that require two or more individuals to work together to achieve a common goal. In these tasks, individuals need to coordinate their actions in time and space, performing identical or different roles, to obtain a reward that can be shared at the end. These are tasks in which individuals' interests are fully aligned because working together is the only way to obtain anything. Normally these are situations in which the information necessary to perform the task is perceptually retrievable and available to all participants, and coordination can be achieved without communication. However, a few studies that are reviewed next have reported communication and attempts to galvanize the partner into action when one of the partners was reluctant to perform her role. The communication strategy covaries with the spatial set-up of the task and involves mostly tactile communication when communicator and recipient were in the same room/space and attention-getters and visual gestures when they were separated in different rooms.

Crawford [44] using a classic heavy box pulling task, reported how two chimpanzees performed soliciting behaviours and gestures (e.g. reaching out the arm, touching and grabbing the partner's shoulder and elbow^3^ towards different partners who refused to pull). These individuals participated in hundreds of trials and there were fluctuations in individuals' motivation towards the food, so that communication appeared when one partner was not interested in pulling. Some individuals may have lost motivation to pull when partners monopolized the scarce and clumped food rewards (two apple pieces) in repeated trials. Similarly, Chalmeau [46] presented to a group of six chimpanzees an apparatus with two distant handles that had to be pulled at the same time. This time the apparatus delivered just a single cherry, making it impossible for individuals to share the food rewards. It was the adult 22-year-old male who monopolized the apparatus and produced most of the operant responses, obtaining nearly all the fruits. He learned to wait for a 2-year-old infant female and pulled whenever she started pulling. Occasionally, tired of waiting for her, he started catching her and bringing her close to the apparatus, a coercive and rather forceful ‘communicative’ strategy. A more recent study also reports how a 24-year-old male recruited and pushed two juveniles (4 and 6 years of age) towards the direction of the buttons of a juice fountain. Individuals could not push and drink at the same time and he successfully coerced the juveniles into pushing for him [45,48]. Similar tactile recruitment or physical manipulation of social partners (i.e. social tool use) has been observed among orangutan mothers, who push and physically manipulate their offspring to get out-of-reach rewards that the mothers can then steal from them [47].

In interactions with humans, there are several observations of chimpanzees soliciting help and trying to physically activate their human partners. Hirata *et al*. [50,51] report how a chimpanzee solicited help from a human partner to pull an out-of-reach baited tray or push a heavy stone by taking the human's hand, while looking at his face and whimpering. Interestingly, the same chimpanzee never solicited help from her conspecific partner, a 7-year-old female who often disengaged from the pulling task. Also, hand-reared gorillas have been observed to employ contact gestures with human caretakers, consisting of grabbing the human hand and directing it to the desired target, which was accompanied by eye contact and attention-checking behaviours [68,69].

Finally, two studies have systematically introduced cooperation breakdowns to facilitate the emergence of communication in chimpanzees. The study reported in [52] tested children and three hand-raised chimpanzees in several joint action tasks with an adult human partner. The experimenter, who was a zoo caregiver, stopped performing her role at specific times to see if individuals would try to communicate in any form to re-activate the partner. Whereas all children communicated at least once, none of the chimpanzees ever communicated. This contrasts with the results from the studies mentioned above [50,51] which found communication with a human partner. One possibility is that the type of relationship with human partners also impacts the likelihood of communication. Future studies should investigate this possibility in more detail. More recently, Voinov *et al*. [49] also introduced a coordination breakdown scenario and found gestural communication among chimpanzees interacting in a two-touchscreen turn-taking game. In this task, chimpanzees are required to send a virtual target from one screen to another using two touch screens for mutual benefit. The critical manipulation was to simulate a coordination breakdown (i.e. one individual would stop sending the target back) and some subjects, not all, gestured and used attention-getters to try to reactivate the partner.

### (b) Asymmetrical attentional focus

In the joint action tasks discussed above, the challenge was to activate a recalcitrant partner who did not perform her role, but in principle all the necessary information was perceptually available to all, and there was only one mutually beneficial outcome possible. However, in coordination dilemmas, there are several possible mutually beneficial outcomes and partners' attentional foci (or preferences) may not be aligned so that the challenge is to coordinate on one of them (asymmetrical attentional focus).

One particularly interesting dilemma presents individuals with a Stag-Hunt scenario, or the dilemma between safety and social cooperation. Pairs of individuals have a choice between hunting alone a lower quality ‘hare’ or hunting cooperatively a higher quality ‘stag’. In this context, communication to help coordinate initiating the joint action, i.e. going for the ‘stag’, would be extremely beneficial. Studies have found that although chimpanzees do not communicate their intentions to go for the ‘stag’, they are still successful by employing a leader–follower strategy [54]. Some individuals take the risk of leaving the safe low-quality option, and once at the ‘stag’, they wait for the partner to join or intentionally communicate if the partner is too slow to follow. The communication observed in this coordination dilemma has been mainly attention-getters such as handclapping, grid-banging and stomping, and it was mostly observed when partners took longer to join [54]. In a follow-up by [56], the risk of leaving the solo option was increased by making this option a higher-quality reward and reducing the visibility between partners. In this scenario, chimpanzees continued communicating with attention-getters but didn't adapt to the more challenging situation with increased communication, which could have reduced the risks of leaving the ‘hare’ (see also [70]).

Finally, there is also evidence for communication in helping tasks in which one individual can help a partner complete an action goal. These are tasks in which one individual wants something and the partner can altruistically, and at low cost, help. In these contexts, a signal is usually produced and if recognized by a recipient it should be responded to with some helpful/cooperative behaviour. Sometimes this takes the form of intention movements, like reaching gestures. Other times it has more indexical forms like pointing, and this tends to lead to significantly higher comprehension challenges. These examples fit both categories ‘common information’ and ‘asymmetrical attentional focus' because it is difficult to discern whether recipients' attention needs to be drawn to the action problem, or recipients need to be nudged into action.

For example, several paradigms have tested chimpanzees' communicative and helping behaviour in tasks where one subject needs a tool or food is stuck somewhere and only a potential helper can help [57–60]. These studies have found that chimpanzees request help from conspecifics, stretching the arm into the partner's room, producing attention-getters like clapping hands or beating the separating panels between rooms and whimpering. The frequency of requests varies with subject and dyads. For example, Yamamoto *et al*. [59] report that in the mother–offspring pairs requests were observed on average in 89% of the trials, whereas in non-kin pairs this happened in only 37.5% of the trials. Dominants exhibited requests toward the subordinate partner in 64% of the trials, whereas subordinates toward the dominant partner in only 11% of the trials. Overall, requests greatly improved the likelihood of being helped, recipients succeeded in obtaining help in 82% of trials with a request versus 23% of trials without a request.

### (c) Asymmetrical knowledge

Only a handful of studies have created collaborative joint action problems in which one of the partners lacked information to perform their role (asymmetrical knowledge), and communication was a prerequisite for success.^4^

Savage-Rumbaugh *et al*. [61] trained two chimpanzees to use a lexigram keyboard that simulated human linguistic symbols. In the collaborative task, one individual had to identify a specific tool to open a reward box and use the lexigram to request it, whereas the partner had to retrieve the specified tool and give it to the requester, who was then able to obtain the food. Both chimpanzees were first trained by humans in both roles, and then paired together to solve the task. From the second day, they were able to succeed. However, once the lexigram keyboard was removed and they were left with their own communication means, they were unable to solve the problem. Bullinger *et al*. [62,63] tested bonobos', orangutans' and chimpanzees' ability to point towards the hiding location of a tool that the human partner needed to retrieve food for them. Overall, subjects indicated the tool location using different gestures including pointing. Bullinger *et al*. [63] added a second condition in which the human experimenter needed the tool to retrieve a reward for herself, and in this purely informative condition, subjects did not point reliably anymore.

Moore *et al*. [72] also tested pairs of orangutans in a task where the communicator could see the location of food but not reach it, and the potential helper could not see where the food was but could release it to the partner. They found that one male orangutan pointed regularly to the food location, but helpers almost never reacted and when they did, not always correctly. However, it is important to note that because recipients didn't get any food, this is a helping task rather than a mutually beneficial joint action task, so the study is not only measuring their comprehension and coordination abilities but also their altruistic motivation (see also [73]).

Melis & Tomasello [64] tested chimpanzees' ability to help their partner perform their role in a collaborative food-retrieval task. The task required pairs of chimpanzees to perform two sequential roles to access rewards from a box. For each role, subjects required a specific, not interchangeable tool. In the test, one individual in each pair was given both tools, and the study found that most subjects helped and transferred the correct tool to the partner. Although the study was not conceived to investigate communication, the first tool transfer in 8 out of 10 subjects was preceded by a request from the partner, who stretched out their hand in the direction of the needed tool. Afterwards, tool transfers occurred without communication. It is possible that requests in this task helped subjects to understand the interdependence of their actions and that by helping their partners they actually helped themselves (in [59,60] responses to requests were altruistic).

Another study [55] specifically tested chimpanzees’ communicative means to provide their partner with information about the location of a tool necessary to solve a mutually beneficial food-retrieval task. However, subjects did not reliably communicate nor comprehend their partner's communicative behaviours. Communicators who knew the location of the tool sometimes positioned themselves in front of the tool location and combined this behaviour with attention-getters (e.g. stomping, jumping, mesh-banging). However, the recipients did not follow these signals so, overall, the dyads did not succeed in obtaining the rewards, and the communicators stopped communicating.

The problem in this study [55] was the recipients. The recipients chose very fast one of the two hiding locations, without paying attention to or ignoring the communicators. Had the recipients even only occasionally followed the communicators' signals, they would have succeeded, and the communicators would have continued communicating, mutually reinforcing each other's behaviour. A more recent study [65] employed a different paradigm and introduced several methodological changes to prevent the recipients from making an impulsive choice. The chimpanzee pairs needed the same two tools as in [64]. The communicator in each pair could see the location of the tools (hidden in one of two opaque boxes), whereas only the recipient could open the boxes. One important change was that the communicators were also in control of the key that recipients needed to extract the tools from the hiding location. The study found that 8 out of 10 communicators increasingly communicated the tools' location, by approaching the location and giving the key needed to open it to the recipients close to the box. The recipient used these signals and obtained the tools, then transferred one to the communicator, and finally, working together with the two tools, the pairs obtained the grapes. The key difference between the two studies [55,65] was that recipients in the latter study paid attention to the communicators, but they were partly forced to, since the communicators also had the key necessary to open the hiding location. In a control condition with no communicator present, but in which the key was placed by the experimenter next to the baited hiding location, they did not perform above chance. One possibility is that when recipients started following the communicator's signals, the communicator's behaviour was positively reinforced, which led to a spiralling of successful production and comprehension of communicative signals. We discuss these findings in more detail below.

## 3. Discussion

### (a) Production

We have reviewed different situations in which communication occurs and facilitates coordination (table 1). They are usually scenarios in which one individual needs assistance that only the other participants can provide (i.e. they cannot complete the task by themselves) and the recipient's attention needs to be directed towards a specific object. There are also scenarios in which the communicator has privileged access to information that the partner has not (e.g. they can see where something is hidden while their partner cannot). Based on the evidence reviewed above, in such scenarios, great apes can communicate to (i) recruit others' assistance, (ii) gather others' attention and (iii) to direct a partner's attention to specific location and action. In other words, from a signal production perspective, great apes are clearly capable of engaging in what others have labelled first-order intentionality (see [9]), i.e. communicators have a specific goal, and they want a specific response from the recipient. It is, however, unclear to what degree they might engage in second-order intentionality and aim to change their recipient's knowledge state.

Among the factors that are often overlooked in these cooperation studies are two that appear to be critical for the occurrence of communication in the first place: spatial arrangement/proximity and relationship between participants. The review of the studies above shows how the specific communication strategies covary with the different spatial arrangements and distances between communicator, recipient and desired object.

On the one side there are observations of visual and acoustic attention-getters, and gestures like pointing in distal set-ups, when partners are separated in different rooms with several barriers between them, and when subjects are physically distant from what they want (e.g. [54,72]). On the other side there are observations of tactile communication and coercive physical recruitment of potential helpers, when partners are in the same space and the recruited partner is an infant or juvenile subordinate individual (e.g. [46,48]). In proximal set-ups that allow communicators to approach and touch the desired object, and when partners are separated but could potentially still touch each other through the separating bars, we have not observed attention-getters and instead observe evidence-rich behaviours such as approaching the desired object, looking at it and ‘offering’ the partner the tool necessary to manipulate it ([65], CJ Völter, E Felsche, J Call, F Rossano 2022, unpublished data).

This evidence is in line with previous ecological explanations about the contextual parameters for pointing behaviour in apes [74,75]. If the object of interest can be approached and manipulated directly this is what apes tend to do, whereas when this is prevented by distance and barriers, they can employ acoustic and visual signals that grab the recipients' attention and direct them to a specific target/location.

There is also tentative evidence suggesting that the relationship and the degree of tolerance between partners has an impact on individuals’ likelihood to communicate. This is not only in line with what is usually assumed for human communication, i.e. an underlying assumption that the partner will be cooperative, but also with several studies on primate communication. Tactile communication and the most coercive physical manipulative strategies have been observed towards infants, juveniles and human partners, all of whom are more likely to respond positively and in a playful manner, or simply ignore the partner, but unlikely to respond aggressively (e.g. [46,50]). Requesting behaviour for a tool, in the form of stretching out the arm into the partner's room while using attention-getters, was much more common among highly tolerant mother–offspring pairs than among non-kin pairs, and from dominant towards subordinate individuals than vice versa among non-kin pairs [59]. As is the case among young individuals, in the same way that higher tolerance levels facilitate the emergence of joint action and cooperation (e.g. [15,71]), it is possible that communication and soliciting behaviour also emerges more easily among highly tolerant partners. In addition, different relationships between partners probably also determines how successful requests are, and individuals may learn through trial and error whom to solicit help from [51,74]. This would explain the frequently observed soliciting behaviours towards human partners, who are generally cooperative and responsive to chimpanzees' requests [50,51,69].

This fits with naturalistic observations showing that food sharing requests are more likely to be successful when occurring between kin (especially mother–offspring) and closely bonded individuals [76,77]. Note that a close relationship implies the likelihood of a more cooperative recipient and maybe even a more attentive recipient, i.e. one more likely to respond to the production of even a subtle communicative signal. This means for example that while one might expect more recruitments [42] and requests to occur between closely bonded individuals, other signals such as attention-getters or manipulating the recipient's attention might be minimized and produced in a less conspicuous and shortened manner (see e.g. [23]). So, the effect of social relationship on the amount, type and complexity of communicative signals is nuanced and further empirical studies will need to investigate this further.

### (b) Comprehension

We have reviewed cases of joint action tasks in which subjects knew what to do but were simply unmotivated or uninterested in the task, and infant or juvenile partners were recruited via tactile communication (including dragging, pushing, e.g. [44,46,48]). We have also reviewed cases of ‘asymmetric attention’ in which the communicators directed the partner's attention towards the task using attention-getters (sometimes accompanied by other gestures), in an effort to make it salient and recruit their help. These were situations in which recipients were familiar with the specific action problem (and communicators' and recipients' preferences were aligned), but had initially not paid attention to it or were focused on something else. For example, in the stag-hunt scenario when ‘leaders’ made salient the possibility of getting the ‘stag’, partners simply followed [54]. This was similar in altruistic helping tasks, once communicators directed the recipient's attention to their problem (e.g. [57,58]) or communicated their more specific need (i.e. stretching out the arm in the direction of the tools in [59] partners often provided the necessary help).

The most challenging cases of communication from a comprehension point of view are those of ‘asymmetric knowledge’ in which recipients cannot fulfil their role without additional information and/or they have to make some inferences about the communicator's goals. For example, Yamamoto *et al*. [60] found that chimpanzees help conspecific partners by giving them the tool they need. Although helping occurred in response to gestural requests, recipients were able to choose the correct tool from an array of tools, but only when they had visual access to the communicator's specific problem task. This suggests that although the requesting gestures were not specific enough, recipients were able to infer the specific communicator's goal.

In some other tasks, recipients were ignorant about the location of the hidden rewards and were dependent on the information provided by the communicators (e.g. [55,65,72]). This situation resembles the typical object-choice pointing studies, in which non-human apes have generally performed at chance levels [78]. There is evidence from at least two studies [55,72] showing gestural communication directed towards the baited location, but where recipients either ignored the communicators [55] or followed the pointing gestures only occasionally (including those of humans [72]). However, recipients in [65] performed above chance levels following the evidence-rich conspecific signals and they were also above chance (80% of the trials) in following distal cross-pointing gestures of a human experimenter.

Despite the positive findings of Melis & Tomasello [65] in which the communicators were able to use more expressive and meaningful behaviours, the existing evidence suggests that in these cooperation tasks, recipients often struggle using the communicator's signals, and this can negatively affect the production of signals so that the communicator would lose motivation to even try to communicate (e.g. [55]). This is in line with the large number of studies that have found that apes do not use pointing gestures reliably (e.g. [78,79]). Nevertheless, there are a handful of studies in which they have performed above chance, and it is important to investigate further the factors that seem to improve subjects' pointing comprehension. Currently, there are four main factors that seem to improve their performance.

Apes raised in a rich social-communicative environment perform significantly better than other apes [80,81]. Local enhancement, in the form of approaching behaviour and sitting close to the target or proximal pointing, can also improve performance [81–83]. A distal object-choice set-up, in which subjects have to approach the chosen location, may facilitate subjects’ attention to the communicators' signals and has also been associated with improved performance [14,65,84,85]. Finally, there is accumulating evidence that adding vocalizations or sounds to the pointing gesture improves performance [65,81,83,86].

One possible explanation for apes’ weaker performance interpreting referential gestures is that they do not understand cooperative communicative intentions. Although they may follow a point and recognize the referent, they are unable to figure out the communicator's helpful intent. Instead, they tend to interpret pointing as ritualistic reaching for the pointer's own benefit, which only in the context of competitive scenarios helps them infer that ‘there must be food there’ [13,14,31]. However, the positive findings from the studies above suggest that under some circumstances they are able to make sense of informative pointing and that this skill is not limited to (strongly) enculturated apes who have been reared in socio-linguistically complex environments (e.g. [81]). There are potentially two main hypotheses that explain their improved performance.

One possibility is that some of the methods employed have been more successful at grabbing the subjects' attention (the ‘attention boosting hypothesis’ [86]). For example, a distal set-up may facilitate their paying attention since the food containers and the communicators' signal are not on the same plane competing for subjects' attention, as is the case in the proximal set-up [65,85]. Similarly, vocalizations and sounds may grab subjects' attention, helping them realize the human informative intention [65,81,83,86]. Performing better in a competitive than a cooperative context could maybe even be attributed to the higher attention-grabbing effect of an authoritative and prohibitive ‘No, don't take this one’ over a friendly ‘Look here’ [14].

Another hypothesis is that the problem is purely one of interpretation and ascribing meaning to the reason for the signal [40]. Pointing and simply referring to an external target is meaningless without some common ground between communicator and recipient, unless the referent is naturally meaningful in its own way, e.g. pointing to a snake you are about to step on. Moore [40] argues that apes are limited in their capacity to track common ground (though see [87]) and that this is the reason for their difficulty making sense of *evidence-poor* signals like pointing (as opposed to their *evidence-rich* gestural communication). His argument is that accompanying points with more naturally meaningful behaviours, such as vocalizations or facial expressions, might help recipients infer the communicator's reason for indicating the referent, which would fit some of the evidence reviewed above.

Note that these hypotheses are not mutually exclusive and might also help us understand why the strength of two individuals' relationship might play a major role in how communication in cooperation tasks might occur and be beneficial. Indeed, between closely bonded individuals, one might expect a higher level of cooperativeness, a heightened default attention and a stronger common ground, facilitating the perception of communicative signals, the inferential processes necessary to decode their meaning and the likelihood to respond to such signals. Future empirical studies should help disentangle whether one of these factors might be more important than the others when contrasted.

## 4. Conclusion and future directions

We have reviewed accumulating evidence showing that great apes can use a variety of strategies, including intentionally communicative ones, to influence cooperative activities (i.e. there is evidence of ample flexibility and high-quality performance from the communicator side). We have also shown that the main constraining factor is on the comprehension/recipient side. It is as yet unclear to what degree the issue concerns assumptions about cooperative intent, general attention or simply limited common ground, and future empirical studies will need to address such hypotheses.

From a comparative perspective, the tentative suggestion here is that what differs in the human ‘interaction engine’ when compared to non-human great apes are the following recipients' features: (i) a generalized motivation to pay attention to communicative signals produced by all conspecifics, i.e. also non-kin and non-bonded partners; (ii) trust that communication will be honest and cooperative (i.e. not competitive and/or deceptive); and (iii) higher motivation to produce responses to communicative signals produced by non-kin and non-bonded partners. From a signaller's perspective, a large amount of flexibility in signal selection and signal calibration appears to be already present in non-human great apes, yet in humans one can observe (i) an improved epistemic ability to compute the common ground and the degree of asymmetric knowledge (see [88]); (ii) the capacity to represent other conspecifics as having different perspectives, knowledge, beliefs from their own; and (iii) a diminished reliance on tactile communication and increased reliance on distal signalling, even when physical contact could be possible, leading to an increased use of attention-getters and vocal communication in general.

More generally, it seems reasonable to hypothesize that once the sheer size of human groups began to grow and intergroup encounters began to intensify, humans experienced an increase in the frequency of ‘asymmetric knowledge’ compared to ‘common information' and ‘asymmetrical attention’ scenarios. Increased numbers of interactions with strangers, and an improved ability to produce displaced references (referring to objects visually not co-present at the time the signal is produced) in turn, would have led to the need for more frequent communicative signals to address the asymmetric knowledge issue (to achieve common ground and facilitate social cohesion). Simultaneously, this would have likely led to improved inferential processing abilities of ambiguous signalling on the recipient's side in an arm's race towards minimizing the amount of communicative signalling needed in each encounter. This is because cognitive inferences are energetically cheap while communicative signalling is costly and potentially risky if detected by unintended recipients.

Concerning the key factors explaining the likelihood and the type of communication occurring during cooperation studies, we propose dedicating more attention to the physical constraints of the study (proximity of the participants, visual access to the apparatus and to the other participant, etc.) and the age and relationship between the two interactants. These appear to be key factors explaining the likelihood and the type of communication occurring during cooperation studies. Future studies on cooperation and coordination that report more detailed accounts of communication are needed to help us better understand the flexibility and constraints of non-human great apes' communication.

Ideally, future experimental studies could employ cooperation paradigms already known to elicit communication (keeping in mind the different types of communication that normally emerge in different spatial set-ups) and manipulate systematically the relationship between dyads to test how kinship, tolerance and dominance influence patterns of communication. We also need more studies to investigate the cause of recipients’ difficulty using the communicators' referential signals. Another interesting avenue for further research is studies that investigate whether communicators adapt to the recipient's knowledge state (as observed in the wild by [89]), communicating less or differently when recipients already have the necessary information to perform their role. This would help us distinguish between imperative and informative motives and establish once and for all whether they can engage in second-order intentionality. Also, longitudinal studies should be conducted on specific dyads to determine non-human primates’ ability to develop specific conventions over time and whether these can be transferable to other tasks once developed.

Ultimately, further research on the use of communication between non-human primates in cooperative tasks should help us assess whether our cooperation needs led to the development of language or whether having language made us better cooperators.

## Data Availability

This article has no additional data.

## Endnotes

1 We use ‘cooperation’ in its broadest sense, meaning all social interactions in which the actor and recipient benefit or only the recipient. Whereas ‘collaboration’ refers to mutually beneficial interactions in which actors work together to achieve a common goal, ‘helping’ behaviour refers to behaviour in which an individual performs an act that benefits another one, where the benefit may come from success at an action-problem or from sharing a valuable resource [24].

2 We use the term ‘joint action’ following [66] which refers to social interactions whereby two or more individuals coordinate their actions in space and/or time to bring about a change in the environment. However, we propose that there can be variation across species regarding the underlying socio-cognitive mechanisms [67].

3 The soliciting gestures varied from reaching out the hands and moving the arms up and down, to crouching and bouncing up and down with flexed legs and touching and grasping the partner by the shoulder and/or elbow to turn her towards the direction of the apparatus.

4 Although [53] asked this exact question, their pulling task was intuitive enough for chimpanzees to succeed. There were probably tolerance constraints affecting performance as shown by [15,71]. If subjects are not even tolerant enough to manipulate the apparatus at the same time, it is also unlikely that they would solicit help from each other.
